# Imaging of Cerebral Iron as an Emerging Marker for Brain Aging, Neurodegeneration, and Cerebrovascular Diseases

**DOI:** 10.3390/brainsci15090944

**Published:** 2025-08-29

**Authors:** Chi-Heng Zhou, Yi-Cheng Zhu

**Affiliations:** Department of Neurology, State Key Laboratory of Complex Severe and Rare Diseases, Peking Union Medical College Hospital, Chinese Academy of Medical Sciences and Peking Union Medical College, Beijing 100730, China

**Keywords:** iron, brain aging, neurodegeneration, cerebrovascular disease, neuroimaging, biomarker, quantitative susceptibility mapping

## Abstract

Iron is critical for brain development, metabolism, and function; however, dysregulated iron disposition contributes to neurological diseases. Many neuroimaging techniques have enabled detection of iron susceptibility, and quantitative susceptibility mapping (QSM) offers a sensitive magnetic resonance imaging (MRI) technique for quantifying brain iron. To elucidate the functional role of cerebral iron and clarify the utility of QSM in establishing iron as a potential biomarker, this review synthesizes cellular and regional behaviours of iron from physiological aging to disease conditions, with a focus on neurodegeneration such as Alzheimer’s disease (AD), Parkinson’s disease (PD), and multiple sclerosis (MS), as well as cerebral small vessel disease (CSVD) as cerebrovascular manifestation. Distinct patterns of iron distribution in deep gray matter and selective cortical regions are associated with motor and cognitive impairment, while the interaction between iron, vascular integrity, and glial function further stresses its pathological relevance. QSM of iron may, thereby, serve as a marker to monitor iron-related disease progression and facilitate intervention. Temporal dynamics of iron in brain pathology remain underexplored, and we emphasized the need for longitudinal mapping and multi-modality biomarker integration. Establishing iron as a clinically relevant imaging biomarker requires continued investigation into its topographical, molecular, and functional correlates across aging and disease trajectories.

## 1. Introduction

Iron is critical for brain development and maintenance, but abnormal iron accumulation in certain brain areas is evident across a range of neurological disorders, associated with increased vulnerability to neuronal injury [1,2]. Neuroimaging evidence from the last century first indicated early iron deposition within the brain [3], with growing evidence continues to support the role of iron in many diseases. Iron has considerable potential as a predictive and monitoring biomarker for many neurological diseases; however, it remains unclear whether excess iron represents an initiating factor or a downstream consequence of neuropathology. Moreover, the temporal and spatial dynamics of iron deposition across healthy aging and disease states are still poorly defined.

**Figure 1:**
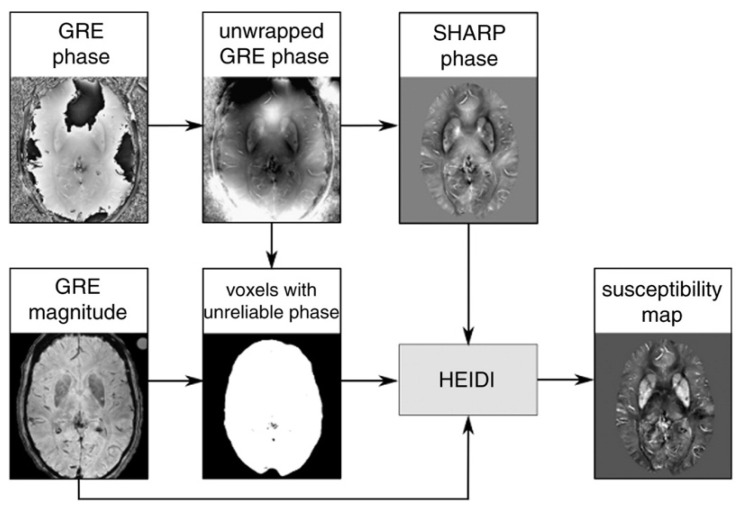
Schematic illustration of the QSM processing using High-resolution Enabled Dipole Inversion (HEIDI). Reprinted from Neuroimage, Vol. 62, Langkammer et al. [4]. Quantitative susceptibility mapping (QSM) as a means to measure brain iron? A postmortem validation study, Pages No. 1593–1599, Copyright (2012), with permission from Elsevier.

Quantitative susceptibility mapping (QSM) is an advanced post-processing technique enabling estimation of the bulk magnetic susceptibility of tissue *in vivo* from gradient echo (GRE) magnetic resonance phase images [4] 29 August 2025 1:44:00 p.m. The standard QSM pipeline involves phase unwrapping, background field removal, and dipole inversion to convert raw MRI phase data into a spatial map of magnetic susceptibility (Figure 1). QSM has emerged as a widely used non-invasive technique for quantifying brain iron content.

The current review summarizes the latest highlights about the relationship among iron metabolism, normal aging, and neurological diseases. It further emphasizes the associations between established neurological biomarkers and iron as measured by susceptibility-sensitive neuroimaging techniques, like susceptibility-weighted imaging (SWI) and QSM, convicting the role of iron as an emerging neuroimaging biomarker for neurological disorders across neurodegenerative, neuroinflammatory, and neurovascular etiology.

## 2. Iron Metabolism in Central Nervous System

Iron-dependent metabolism within the central nervous system (CNS, Table 1) includes oxygen transportation, myelin production, neurotransmitter synthesis, neuronal growth, and energy homeostasis. Two transport iron forms (i.e., transferrin-bound and non-transferrin-bound) and several vital proteins interplay to aid the brain in acquiring and exporting iron. There is also a limited amount of erythrocyte-derived iron entering brain via choroid plexus stromal macrophages, which is evident in hemoglobin-induced post-hemorrhage cephalic injuries [5] such as hydrocephalus [1].

Abundant expression of transferrin receptor 1 (TfR1) on the luminal side of microvascular endothelium allows ferric iron-bound transferrin (holo-Tf) circulated in the periphery to cross the blood–brain barrier (BBB), followed by clathrin-mediated endocytosis [6,7]. Upon the detachment of ferric iron from holo-Tf, six-transmembrane epithelial antigen of prostate 3 (STEAP3) facilitates the reduction in ferric iron into ferrous iron, after which the divalent metal transporters (DMT1) within the BBB endothelial endosomes transfer ferrous iron into the endothelial cytosol [8]. By far, the TfR1-dependent holo-Tf iron uptake is recognized as the canonical route of non-heme iron to cross the BBB and enter the CNS. Non-transferrin-bound iron (NTBI) can also take another route mediated by DMT1 to enter the brain microvascular endothelial cells across the luminal membrane [9]. Meanwhile, DMT1 aids iron uptake from the duodenal epithelium into the bloodstream to satisfy the systemic iron demand [10]. Overall, plenty of the cytoplasmic iron is sequestered in ferritin, and degradation of lysosomal ferritin can build up iron-rich hemosiderin. Interestingly, ferritin and hemosiderin can be detected differentially by susceptibility-sensitive magnetic resonance imaging (MRI) techniques [11], probably because that water-soluble ferritin induces both T1 and T2 shortening, whereas water-insoluble hemosiderin, with larger iron aggregates, causes pronounced T2 shortening without significant T1 effects. Relative contrast ratio analyses have shown that hemosiderin is best detected on T2*-weighted GRE, while ferritin-associated contrast is more prominent on T2-weighted spin echo [12]. Although qualitative differentiation between ferritin and hemosiderin appears feasible, its specificity remains limited without quantitative validation. Current *in vivo* neuroimaging methods, including QSM, lack sufficient reliability to accurately distinguish molecular iron forms.

In addition to DMT1, another protein expressed rich on the brain microvascular endothelium, ferroportin 1 (FPN1), functions as an iron exporter that helps maintain intracellular iron homeostasis [13]. Recent evidence indicates paradoxical roles of brain FPN1 in ischemic stroke. FPN1 deletion reduces iron deposition and infarct volume during the acute phase, yet attenuates the neuronal recovery phase in later stages [14]. On the other hand, diminished FPN1 expression and the resulting iron overload have been consistently linked to neurodegenerative diseases. These findings underscore the context-dependent functions of FPN1 and highlight the need for therapeutic strategies that balance its protective and detrimental effects across disease stages.

Yet, TfR1 complex for iron acquisition and DMT1 for iron recycling represent core pathways for brain iron transportation, although additional proteins such as lactoferrin are recognized in neuronal iron acquisition [15]. Further exploration of neuronal iron metabolism should elucidate how homeostasis is maintained or disrupted, with susceptibility-sensitive neuroimaging providing complementary *in vivo* insights to laboratory findings.

## 3. Glial Loading of Iron

Spare of neurons, the rest of the CNS comprises glial cells with greater demand for iron. Glial cells also employ more complicated regulatory mechanisms to manage iron metabolism, subsequentially fluctuating the general CNS iron levels.

### 3.1. Oligodendrocytes

Oligodendrocytes produce myelin in the CNS and have an ultrahigh demand for iron for its functional maintenance, disruption of which can lead to postnatal hypomyelination and demyelinating disorders like multiple sclerosis (MS) [16,17]. For decades, it was believed that oligodendrocytes do not express the TfR family as neurons. Instead, ferritin heavy chain (H-ferritin) serves as the primary iron provider for oligodendrocytes, and T-cell immunoglobulin mucin domain 1 protein (Tim-1) is the H-ferritin receptor on the membrane of oligodendrocytes [18,19]. Later studies demonstrated that oligodendrocyte progenitor cells indeed express TfRs, which underwent downregulation throughout oligodendrocyte maturation [20]. This TfR cycle is critical for establishing iron homeostasis and ensuring proper postnatal myelination, suggesting that impaired prenatal iron uptake machinery may disrupt oligodendrocyte development and function.

While oligodendrocytes may have different iron demands for metabolism and myelination, the underlying mechanisms and their threshold for pathological iron accumulation remain unclear. Quantitative imaging studies highlighted a robust negative correlation between iron and myelin content in cognitively unimpaired aging individuals [21]. However, longitudinal population studies that simultaneously track cognition, oligodendrocytic iron, and myelin integrity are still lacking.

Recent integrative work has mapped iron- and myelination-relevant gene expression to QSM signals across functionally distinct deep gray matter regions, using six post-mortem brains (age 24–57) and ten healthy adults (age 41–49) [22]. In short, QSM signals positively correlate with ferritin, transferrin, FPN1, and DMT1 expression, reinforcing the link between iron transport activity and tissue susceptibility in deep gray matter [4]. Several oligodendrocyte-associated myelination gene markers also align with high QSM values, consistent with histological evidence showing oligodendrocytes as the predominant cell type in iron-rich regions. The weak correlation between TfR expression and QSM suggests that TfR may primarily mediate BBB import of iron rather than parenchymal accumulation; moreover, TfR absence in mature oligodendrocytes may further account for such limited association. These results underscore that gene-level regulation of iron handling directly determines QSM contrast, with oligodendrocytic iron metabolism as a key contributor and potential driver of myelination deficits and iron-related pathology.

### 3.2. Astrocytes

Being the most plentiful cells in the CNS, astrocytes are closely related to neurons and cerebral micro-vessels, forming neurovascular units, which promise astrocytes a prominent role in mediating vascular cognitive impairment and dementia [23]. Astrocytes utilize a classic Tf iron uptake cycle with TfR1 and DMT1 as neurons but are not eager for iron as neurons or oligodendrocytes [2]. However, given that astrocyte end-foot processes obtain iron through the BBB microvascular endothelial cells and distribute it into the cerebral parenchyma, astrocytic iron is necessary for CNS function. Relating to mature myelinating oligodendrocytes, knockout of H-ferritin from astrocytes led to a remarkable delay of postnatal development of oligodendrocytes and, therefore, a consequent decrease in the number of myelinated axons during both myelination and post-damage remyelination [24]. Moreover, blocking astrocytic expression of DMT1 or TfR1 yields profound thinness of the general cortex. It further demonstrates a neurodevelopmental insufficiency due to astrocytic iron disturbance, and future studies should also explore astrocytic possibilities for myelin rehabilitation.

Mechanisms of how astrocytic iron abnormality contributes to various neurological diseases are still under investigation. In response to high intracellular iron load, astrocytes secrete hepcidin to downregulate FPN1 expression in the brain microvascular endothelium, decreasing iron uptake across the BBB [25]. Due to structural characteristics, there can been therapeutic chances to target astrocytes and associated synapses. Scattered evidence indicates that neuronal electrophysiological tangles and astrocytic iron may function reciprocally [26], suggesting a possible iron chelator therapy to reduce episodic reoccurrence of epilepsy and minimize iron accumulation in neurons and astrocytes involved. Currently, susceptibility-sensitive neuroimaging studies that visualize the spatial and temporal dynamics of astrocytic iron alterations in neurological disorders remain limited. Such investigations are essential to enable preclinical monitoring of iron-related demyelination and may hold prognostic value for neurological outcomes associated with neurovascular dysfunction.

### 3.3. Microglia

Microglia are brain-resident glial cells of yolk sac-derived macrophagic lineage, whose function encompasses regulation of innate immune responses and inflammatory signaling, facilitation of axonal regeneration, and remodeling and repair of disrupted synaptic networks. Like astrocytes, microglia acquire iron through the Tf-TfR pathway in conjunction with DMT1, and export iron via FPN1. Distinct from other glial populations, microglia serve as resident immune cells of the CNS, highlighting a critical interface between iron homeostasis and neuroinflammatory responses.

Microglia constitutively express low levels of heme oxygenase-1 (HO-1), an enzyme essential for neuroprotection during neuroinflammatory and neurodegenerative progresses. However, aberrant overexpression of HO-1, particularly in the aging brain, can paradoxically contribute to neurotoxicity through iron overload [27,28]. Aging is a stronger predictor of microglial HO-1 overexpression than neuroinflammation alone; nevertheless, HO-1 blockade may confer neuroprotection against inflammation-induced iron accumulation, independently of FPN1 or DMT1 regulation [27].

In summary, glial cells act as major CNS iron reservoirs, with uptake mediated by TfR1/DMT1 and export via FPN1 to maintain homeostasis. Disruptions in these pathways can impair myelination, neurovascular support, and immune regulation, thereby contributing to neuropathology. Understanding glial iron loading through susceptibility-sensitive neuroimaging holds promise for identifying early dysfunction and guiding therapeutic strategies.

## 4. Neuroimaging of Brain Iron Distribution in Normal Aging

Early MRI studies have shown that T2-weighted hypointensity correlates with iron distribution [3]. Subsequently, the R2* relaxation rate emerged as a more sensitive linear measure of tissue iron concentration based on theories of susceptibility-induced decay [29]. Nowadays, qualitative SWI and quantitative QSM are particularly notable for their clinical applicability in delineating cerebral susceptibility across diverse conditions, including intracranial hemorrhage, traumatic brain injury, vascular-endothelial disruption, and neurodegeneration [30,31,32]. SWI enhances contrast between tissues with different magnetic susceptibility, enabling visualization of brain iron and structural differentiation, such as gray-white matter boundaries [31]. Compared with SWI and R2*, QSM offers more accurate and reproducible quantification of tissue magnetic susceptibility while capturing spatial distribution of brain iron. Because susceptibility reflects intrinsic molecular and cellular properties, QSM allows more specific discrimination of normal versus pathological regions. Importantly, while vascular calcifications and cerebral microbleeds (CMBs) both appear hypointense on SWI [33,34,35], QSM has overcome this limitation by distinguishing polarity. Calcifications show negative diamagnetic susceptibility, whereas CMBs display positive paramagnetic susceptibility. Such polarity-based separation improves differential specificity, even for small calcifications mimicking CMBs, and enhances the diagnostic value of QSM in aging-related brain changes [36].

During normal aging, brain iron exhibits a region- and cell type-specific manner, predominantly bound to ferritin, hemosiderin, or neuromelanin [37]. Aging is associated with increased vulnerability to neurological disorders, and the mechanisms underlying the shift from healthy to pathological aging remain unclear. Iron may play a role in such transition [38]. Early studies comparing autopsy histochemical staining with T2-weighted MRI revealed age-related brain iron accumulation beginning as early as six months and continuing to over 70 years [3], with negligible iron detected at birth. Initial iron deposition was observed in globus pallidus by 6 months of age, followed by the reticular substantia nigra (SN, 9–12 months), red nucleus (RN, 18 months-2 years), and dentate nucleus (DN) by early childhood up to 7 years. Although iron accumulation plateaus during late adolescence, it substantially increases from the late 60 s onward.

For *in vivo* assessment of iron distribution with simultaneous cognitive evaluations, susceptibility imaging offers better solution and display strong concordance with post-mortem findings and *ex vivo* gene expression profiles [29,39]. Recent QSM studies have demonstrated that subcortical gray matter structures, particularly the basal ganglia, exhibit the highest iron concentrations during normal aging [37,40]. Such spatial alterations predict motor and cognitive decline and may serve as early indicators of neurodegenerative risk. So far, many QSM studies have defined regions of interest (ROIs) based on pathophysiological consensus without first performing voxel-wise whole-brain analyses [41], raising the risk of overlooking iron changes outside predefined areas. This issue is especially relevant given the heterogeneity of aging and the potential for iron involvement beyond classic pathological regions. Advances in whole-brain QSM have allowed a more comprehensive assessment of iron distribution [30], confirming that deep gray matter, including the striatum, SN, RN, subthalamic nucleus, mammillary bodies, and DN, display notable age-related iron accumulation. Among these, SN, RN, and DN appear most affected, consistent with their vulnerability in neurodegenerative disorders.

Cortical iron deposition likewise shows age-dependent variations [38]. Voxel-based whole-brain QSM analyses have demonstrated age-related cortical iron accumulation across functionally relevant regions. In 116 healthy individuals (young group, n = 48, age 20–53; older group, n = 68, age 59–79), prominent susceptibility increases were detected in the superior premotor cortex, prefrontal cortex, insula, dorsomedial frontal cortex, cingulate sulcus, and cerebellar cortex [30]. Similarly, in other 95 healthy participants (age 21–58), significant age-related iron accumulation was observed in motor (precentral, postcentral, premotor), cognitive (prefrontal cortex, superior temporal gyrus, insula, precuneus), and visual (occipital gyri, cuneus, fusiform, calcarine, lingual gyri, posterior cingulum) cortices [42]. Although less pronounced than in deep gray matter, cortical iron accumulation should be given equal priority in biomarker development, as it affects regions critical for fluid cognitive and motor functions [43] and can disrupt essential daily activities [44]. Accordingly, future studies integrating QSM with task-based functional MRI (fMRI) paradigms may provide valuable insights into the neurofunctional consequences of iron accumulation. Cognitive paradigms targeting fluid cognition such as working memory, inhibitory control, and sensorimotor integration, may help determine whether increased iron burden leads to compensatory neural recruitment or diminished processing efficiency, thereby advancing our understanding of iron’s role in adaptive neural control across the lifespan.

Lastly, sex differences in iron deposition across aging have been recognized. Metabolic studies show that serum ferritin rises sharply during the menopausal transition and continues to increase after the final menstrual period in healthy women without malabsorption [45,46], implying a potential risk of heightened brain iron deposition in the postmenopausal period. In fact, *in vivo* QSM has demonstrated lower susceptibility in SN of women after adjusting for age [47]. Whereas men exhibit a linear age-related increase in pulvinar iron, women reach a plateau from midlife onward, and postmenopausal women display lower total subcortical susceptibility compared with both younger women and men of any age. Conversely, men show a synchronized pattern of ferritin and testosterone changes, which has been linked to greater brain iron accumulation and increased risk of neurodegenerative transitions [48]. Together, these findings conclude a sex-related divergence between peripheral iron markers and neuroimaging measures, underscoring the need to disentangle systemic and brain-specific mechanisms of hormone-dependent iron regulation.

## 5. Imaging Iron in Diseases and Correlation with Known Biomarkers

Beyond normal aging, the progressive and irreversible nature of many neurological disorders poses major challenges for preclinical intervention. Extensive proteometabolic and genomic research has sought to identify reliable biomarkers of disease progression in parallel with neuroimaging studies. Establishing concordance between neuroimaging features and molecular biomarkers in specific brain regions is, therefore, critical for enhancing diagnostic precision and advancing clinical translation. A key research priority is to clarify the role of iron deposition in neurofunctional impairment, supported by both qualitative and quantitative neuroimaging evidence.

### 5.1. Parkinson’s Disease

Parkinson’s disease (PD) is a neurodegenerative disease characterized by both motor and non-motor symptoms, including bradykinesia, rigidity, tremor, gait difficulties and impairments in neuropsychological functions [49]. PD is also one of the most extensively studied α-synucleinopathies in relation to neurodegenerative iron deposition, where α-synuclein is an aberrant presynaptic protein forming toxic oligomers and Lewy bodies in PD.

Regarding the central feature of PD pathology, SN, the major site of dopamine (DA) synthesis and regulation, undergoes severe degeneration in PD. SWI and QSM studies have consistently identified SN as the site of robust iron accumulation in PD, with total iron increasing along disease progression [50,51,52]. Because iron serves as a cofactor for tyrosine hydroxylase, a rate-limiting enzyme in dopamine synthesis [53,54], degeneration of dopaminergic neurons may drive compensatory upregulation of DA production in surviving neurons, thereby increasing iron recruitment within the SN.

Serial DA transporter imaging has discovered nigrostriatal dopaminergic dysfunction in individuals with idiopathic rapid eye movement sleep behaviour disorder (iRBD), a recognized prodromal predictor of α-synucleinopathies [55]. QSM of nigrosome (N1), a dorsolateral SN pars compacta (SNc) subregion rich in pigmented dopaminergic neurons, shows slightly elevated iron content in iRBD, with progressive increases reaching substantial levels by the time of PD diagnosis [56]. Although N1 iron accumulation is not yet established as a marker of early PD, it may precede the onset of motor symptoms, underscoring its potential for preclinical intervention and the importance of examining iron distribution across SN subdivisions. Furthermore, iron distribution within SNc may vary by cell type: astrocytic iron stays relatively stable, oligodendrocytic ferritin markedly decreases, while neurons predominantly accumulate iron [57]; these observations remain supported primarily by histological evidence. Future studies should attempt to combine cellular-level iron mapping with other biomarkers to elucidate the mechanistic interplay between iron and PD pathology.

Beyond SN, latest longitudinal work indicates that QSM across multiple brain regions holds prognostic value for cognitive and motor decline in PD [58]. In this study, ROIs were selected from regions showing the strongest voxel-wise associations with clinical outcomes and included structures central to PD pathology, including SN, RN, DN, basal ganglia, hippocampus, nucleus basalis, insula, and orbitofrontal cortices [58,59]. In a 3-year follow-up of 59 PD patients without baseline dementia and 22 controls, higher baseline susceptibility in the temporal cortex, nucleus basalis, and putamen predicted greater future cognitive severity, while elevated susceptibility in basal ganglia, RN, insula, and DN predicted worsening motor outcomes. Additional hippocampal susceptibility increases at follow-up were also linked to cognitive decline. These findings support QSM as a tool for detecting early iron deposition preceding overt PD symptoms. Nevertheless, all follow-up assessments were conducted under ongoing L-dopa treatment, which in mice has been shown to modulate brain iron levels [60] and mask motor severity. Future human studies should therefore examine how PD treatment influence QSM patterns, thereby clarifying therapy-related effects on iron deposition.

In summary, current evidence indicates that iron accumulation in SN closely parallels dopaminergic degeneration, with iron overload observed in both symptomatic and prodromal PD across multiple brain regions. It remains uncertain whether iron deposition drives α-synuclein aggregation and dopaminergic loss, or whether it primarily represents a downstream by-product of neuronal degeneration. Cellular heterogeneity adds further complexity. Iron is therefore best regarded as both a potential amplifier of α-synuclein toxicity and a by-product of dopaminergic failure, underscoring the need for longitudinal and cellular-level QSM studies integrated with complementary biomarkers.

### 5.2. Alzheimer’s Disease 

AD is a leading cause of clinical dementia. Pathological lesions may be present long before symptom onset, with mild cognitive impairment (MCI) representing an intermediate stage preceding overt dementia, stressing the prolonged preclinical phase of the disease and space for early intervention [61]. Efforts to enable early AD diagnosis include the identification of genetic risks and novel biomarkers across molecular, blood-based, and neuroimaging domains. Iron has also recently emerged as a promising neuroimaging biomarker for AD, demonstrating consistency in relation to existing biomarkers.

In patients with MCI and AD, significant iron deposition was observed in the pallidum, caudate, and putamen, with the latter two exhibiting uneven spatial distributions [51,62]. A substantial discrepancy between MCI and AD iron further suggested a progressive increase in iron deposition across disease stages, similar as observed in iRBD and PD. SN iron concentration in AD patients also surpassed that of healthy old adults, indicating a shared vulnerability to iron overload in the SN region across different neurodegenerative pathologies.

Extracellular β-amyloid (Aβ) aggregates into neuronal plaques that disrupt signaling communication in AD, typically identified through immunohistochemistry or positron emitting tomography (PET) imaging. Some researchers proposed that glial iron exacerbate Aβ formation [63], while others suggested that Aβ plaques may facilitate iron accumulation at first [64]. Interestingly, iron was observed within Aβ plaques in both rodent and human tissues via R2* relaxation imaging, raising questions about whether iron or the amyloid presence contributed to transverse proton relaxation rates [65], although species differences complicate translation. *In vivo* QSM has discovered abnormal tissue susceptibility correlated with elevated amyloid PET uptake ratios in the globus pallidus and putamen among patients with MCI and AD [62,66]. Elevated iron deposition was also found in the hippocampus, particularly in the hippocampal fimbria, associated with amyloid plaques in adjacent white matter [67].

Cross-sectional voxel-based QSM analysis of 150 cognitively normal participants (mean age = 69 ± 8 years; 97 completed amyloid PET) from the Biomarkers for Older Controls at Risk for Dementia (BIOCARD) study further showed that iron in the hippocampus, frontal cortex, and deep gray nuclei predicted episodic memory and executive dysfunction in both PET-Aβ-positive and PET-Aβ-negative participants, whereas Aβ alone was not significantly associated with global cognition [66]. To date, no longitudinal QSM-PET studies have tracked initially Aβ-negative individuals to capture conversion and define iron–amyloid dynamics. Similarly, whole-brain longitudinal QSM studies across the MCI–AD trajectory remain scarce.

Taken together, converging evidence indicates that iron accumulation in deep gray nuclei and hippocampus co-occurs with amyloid plaques in MCI and AD, predicting cognitive decline, although independent effects are also evident. The unresolved directionality of the iron-Aβ relationship highlights the need for longitudinal and experimental studies integrating QSM, PET, and complementary fMRI to clarify their dynamic interplay and to assess potential differences in neural activation patterns linked to iron-Aβ distribution.

### 5.3. Multiple Sclerosis

Multiple sclerosis (MS) is a chronic neurodegenerative autoimmune disease characterized by inflammation and myelin destruction, often leading to severe disability. MS presents high heterogeneity in radiological, pathological, and prognostic manifestations and continues to lack reliable, sensitive, and specific biomarkers for consistent clinical monitoring [68,69]. Although biomarkers such as oligoclonal bands, immunoglobin G (IgG) index, and aquaporin-4 (AQP-4) are currently used to assess MS activity and treatment response, no key biomarkers like α-synuclein in PD or Aβ in AD have been established for MS [70]. Given the glial iron reservoir, increasing attention has focused on the contribution of iron to demyelination, and iron quantification has been proposed as a biomarker to improve MS monitoring.

Comparable studies employing R2* and QSM revealed elevated paramagnetic susceptibility in basal ganglia in both clinically isolated syndrome suggestive of MS and confirmed MS patients [71]. Notably, individuals with suggestive MS already exhibited significantly higher susceptibility in the caudate and putamen, pointing to early-stage alterations. Unlike other disorders where susceptibility changes largely reflect iron accumulation, demyelination itself can significantly influence QSM signals in MS. As discussed in the context of glial iron, oligodendrocyte damage and microglial activation are thought to drive iron release and redistribution throughout the course of MS. The liberated and deposited iron can then influence demyelination, chronic inflammation, and remyelination [27,72]. Histopathological studies have confirmed stage-dependent variations in MS lesions that closely parallel QSM findings, supporting that QSM is more sensitive than R2* in detecting susceptibility alterations, though the exact sources of its sensitivity remain incompletely understood [73,74,75].

In addition to deep gray matter involvement, thalamic atrophy was reported in MS patients with substantial physical disability [76], yet opinions on thalamic iron remain inconsistent, with both increases and decreases described [77,78,79]. These discrepancies may reflect heterogeneity in MS stage among study populations and variations in neuroimaging protocols, proposing the value of QSM for more precise assessment of region-specific iron–clinical relationships. Lesion-level QSM has further demonstrated distinct susceptibility patterns. Chronic inactive plaques appear hyperintense, characterized by extensive axon damage and ongoing myelin loss [74]. Chronic active lesions display a peripheral rim of elevated susceptibility attributed to activated microglia at the edge and diminished iron-laden glial cells in the lesion core. Fully remyelinated areas appear hypointense, reflecting preserved myelin [73].

Overall, QSM consistently reveals elevated susceptibility in the basal ganglia and at lesion rims, connecting iron metabolism to demyelination and MS lesion evolution. One interpretation is iron toxicity, in which excess iron drives oxidative stress and lipid peroxidation, rendering the lipid-rich myelin vulnerable. Alternatively, the association may indicate redistribution of iron following oligodendrocyte degeneration. Thus, iron accumulation in MS likely represents both a secondary marker of glial pathology and an active modulator of disease progression. As MS advances, iron levels in deep gray matter can rise, but changes due to comorbid vascular lesion or broader CNS inflammation may confound the interpretation of MS-specific alterations [80,81].

### 5.4. Cerebral Small Vascular Diseases

Cerebral small vessel disease (CSVD) affects brain arterioles, capillaries, and venules, resulting from miscellaneous pathogenetic factors [82]. It is a major contributor to age-related neurological disorders such as stroke, dementia, and gait impairment, associated with poor prognosis. Established MRI features associated with CSVD include white matter hyperintensities (WMH), cerebral microbleeds (CMB), lacunes, enlarged perivascular spaces (EPVS), and global brain atrophy. A recent QSM study suggests that BBB disruption, a hallmark of small vessel pathology, may manifest as iron accumulation [83], with correlations between iron content and established CSVD markers [84] further support iron as an emerging indicator of CSVD burden.

CMBs elicit dual contribution to cerebrovascular and neurodegenerative processes. QSM shows that CSVD patients with prominent CMBs exhibit significantly higher susceptibility in putamen, thalamus, and hippocampus, accompanied by poorer Montreal cognitive assessment (MoCA) and executive function scores compared to CMB-negative CSVD patients and controls [84]. These findings align with evidence from neurodegenerative cohorts, suggesting that basal ganglia iron may exacerbate cognitive impairment in the presence of microvascular lesions. CSVD involves direct vascular-structural injury that can promote iron leakage independent of neurodegenerative dysregulation. In cerebral autosomal dominant arteriopathy with subcortical infarcts and leukoencephalopathy (CADASIL), a monogenic CSVD subtype with *NOTCH*3 mutation, iron accumulation has been mechanistically linked to vascular disruption [83]. In this study, 11 asymptomatic and 10 symptomatic *NOTCH*3 mutation carriers (mean age = 51.1 ± 14.4 years) and 21 matched controls underwent 3T MRI scan. ROI analysis incorporated both voxel-based QSM clusters associated with BBB permeability and theoretically iron-rich structures. Greater QSM values in symptomatic subjects in putamen, caudate nucleus, temporal pole, and centrum semiovale correlate with MRI burden of BBB damages and poorer cognitive outcomes. Advanced field strength may further enhance QSM as a detection tool for CMBs. In a study of 48 participants (mean age = 70.9 ± 8.8 years; 6 with probable cerebral amyloid angiopathy, 9 with mixed CSVD, 2 with hypertensive arteriopathy, and 31 controls), 7T QSM detected more microbleeds than 3T T2*-weighted MRI [85], although a direct comparison of QSM reconstructed from 3T versus 7T images in CSVD remains lacking.

In conclusion, QSM holds promise for detecting vascular lesions and predicting long-term outcomes in sporadic and monogenic CSVD. Due to the cross-sectional design of existing studies, there remains a tendency, but insufficient evidence, to determine whether iron deposition initiates microvascular injury or arises secondarily from BBB disruption. Longitudinal investigations are needed to further clarify the temporal dynamics of iron in CSVD pathogenesis, and future research should also account for the potential influence of other paramagnetic substances on QSM signals. As CSVD is a highly prevalent age-related condition without pathognomonic deposits such as Aβ or α-synuclein, comorbidities warrant particular attention. Beyond aging itself, both diabetes and smoking have been associated with increased basal ganglia susceptibility [86], underscoring the need for future studies to adjust for these factors. See Table 2 for a summary of QSM findings across normal aging and disease, highlighting progressive mapping, multimodal concordance, and unresolved questions. See Table 3 for an overview of regional iron distribution patterns in normal aging and disease profiles.

## 6. Discussion

Iron is essential for CNS development and metabolism; however, excessive accumulation is neurotoxic and has been implicated in the pathogenesis of various neurological conditions. To maintain cerebral and BBB homeostasis, neurons and glial cells regulate iron uptake and export through distinct yet interconnected pathways. Disruptions in such regulatory mechanisms can lead to abnormal iron deposition, which may contribute to age-related changes and precede clinically evident neurological manifestations. Of particular interest, glial iron represents a promising therapeutic and rehabilitative target because of its broad involvement in neurovascular maintenance, immune regulation, and myelin integrity.

Iron deposition begins in infancy and progresses throughout life, peaking in later adulthood. Deep gray matter structures such as the SN, RN, putamen, globus pallidus, and DN show the most pronounced age-related accumulation. These nuclei, along with cortical regions, correlate with motor and cognitive decline, and even asymptomatic individuals with elevated iron carry increased risk of future neurodegeneration and cerebrovascular disease. Post-mortem histopathological and conventional neuroimaging studies demonstrated the topographical distribution of iron in relation to functional deficits. QSM, in particular, provides precise quantification of tissue susceptibility contrast, distinguishing physiological from pathological iron accumulation and thereby serving as a valuable imaging biomarker for brain aging, neurocognitive impairment, vascular pathology, and neuroinflammation.

Several critical questions require further investigation to ensure the clinical validity of QSM iron measurements. First, the temporal and causal relationship between QSM-derived iron and molecular or genetic biomarkers (e.g., Aβ, α-synuclein) is incompletely defined. Whether iron accumulation precedes, co-occurs with, or follows other pathological burdens remains uncertain, complicating causal inference. Similarly, in common cerebrovascular diseases such as CSVD, it remains ambiguous whether iron contributes to endothelial injury and BBB disruption or reflects downstream effects of vascular pathology. If iron proves to be an initial contributor to vascular dysfunction, its quantification could serve as a sensitive preclinical biomarker for identifying individuals with genetic predisposition or comorbid conditions at risk of stroke and vascular dementia.

Second, although recognition of iron as both a driver and a by-product of neuropathology opens new therapeutic avenues, its clinical implications remain debated. Iron chelators such as deferiprone and deferoxamine reduce brain iron in PD and AD [87,88]; however, their impact on disease deterioration remains highly controversial [89,90]. It thus highlights the need for standardized regimens of chelator treatment, as well as a deeper understanding of iron’s role in neurodegeneration. Modulation of systemic and cellular iron homeostasis through agents targeting FPN and TfRs may represent future strategies to restore iron balance without disrupting physiological functions. Antioxidants and ferroptosis inhibitors may provide complementary benefits by counteracting oxidative stress and lipid peroxidation triggered by iron overload [91]. In CSVD, strategies aimed at reducing iron-induced BBB injury or enhancing glial buffering capacity could be integrated with conventional vascular risk management. Importantly, QSM offers a non-invasive tool to guide therapeutic monitoring by quantifying iron burden before and after intervention, thereby enabling patient stratification and individualized treatment.

Third, despite the clinical potential of QSM and the need for longitudinal studies to track iron trajectories in neurological disease, standardization and rigorous evaluation of reproducibility remain essential across multicenter and multivendor settings, as well as within-subject repeatability [92,93,94]. Initiatives, including the RIN–Neuroimaging Network [95,96] and QSM consensus organization [97] have advanced the harmonization of acquisition and reconstruction pipelines, while QSM Challenge committee continues to benchmark and compare reconstruction algorithms [93].

Several methodological recommendations have emerged. QSM results remain highly sensitive to parameters such as field strength, echo time, spatial resolution, and head orientation. Most studies discussed in the current review have used 1.5T, 3T or 7T MRI. While susceptibility estimates can be aligned across field strengths with optimized echo times (TE) [98], inter-scan repeatability improves when reconstructions use 7 or 8 echoes, with intra-class correlation coefficient (ICC) values ≥ 0.75 across most brain regions [99]. 7T MRI enhances basal ganglia reproducibility relative to 1.5T or 3T, though cortical improvements are modest [94]. Among reconstruction methods, Calculation Of Susceptibility through Multiple Orientation Sampling (COSMOS) [100] effectively reduces orientation-dependent errors but remains impractical for clinical and longitudinal use due to complexity and acquisition demands. While no single algorithm can yet be recommended as gold standard, results from QSM Challenge 2.0 suggest that iterative methods generally outperform deep learning approaches, which in turn yield fewer errors than direct inversion techniques under comprehensive evaluation, offering preliminary guidance for latest clinical applications.

Finally, methodological challenges such as the partial volume effect remain critical, which is originally recognized in PET tumor imaging [101]. It arises when a single voxel contains a mixture of different tissues, causing their signals to be averaged and resulting in biased or inaccurate susceptibility estimates. In venous QSM studies, strategies such as model-based corrections and erosion for single-vein segmentations have been applied to large sinuses to mitigate partial volume effects [102]; however, these methods are impractical for smaller veins and nuclei, where erosion would remove most voxels and preclude reliable analysis. Moreover, ROI definition depends on atlas choice and segmentation methods, with inconsistent anatomical boundaries leading to variability across studies. Higher spatial resolution, probabilistic tissue modeling, and harmonized atlas-based definitions should therefore be prioritized in future multicenter work. Ultimately, cautious methodological design and standardization are crucial to establish QSM as a robust clinical tool for iron biomarker application.

## 7. Conclusions

In conclusion, brain iron accumulation emerges as a pivotal yet modifiable factor in brain aging, neurodegenerative progression, and cerebrovascular pathology. By offering a non-invasive, sensitive, and quantitative assessment of tissue susceptibility, QSM provides unique advantages for detecting and tracking iron-related abnormalities across the continuum from healthy aging to overt neurological diseases. Moving forward, integrating QSM with molecular and genetic biomarkers will be critical to ensure reproducibility and clinical translation. Finally, multimodal and longitudinal study designs are needed to elucidate the mechanistic pathways, spatial dynamics, and functional consequences of excessive iron deposition in the CNS, ultimately advancing QSM toward routine use as both a biomarker and a therapeutic monitoring tool.

## Figures and Tables

**Table 1:** Summary of mechanisms regarding iron metabolism in CNS cells.

Cell Type	Iron Uptake	Iron Export	Physiological Relevance	Pathological Relevance
Neurons	TfR1-mediated holo-Tf uptake; DMT1-mediated NTBI transport	FPN1-mediated	Neurotransmitter synthesis, oxygen transport, energy metabolism	Iron overload → Oxidative stress, excitotoxicity, neuronal death
Oligodendrocytes	H-ferritin via Tim-1 receptor; Progenitors express TfRs	FPN1-mediated	Myelination, axonal support	Iron dysregulation → Hypomyelination, demyelination (e.g., MS); Degenerated oligodendrocytes → Released iron can deposit
Astrocytes	TfR1/DMT1 uptake; End foot uptake from BBB endothelium	FPN1-mediated	Neurovascular unit maintenance, myelination support, metabolic buffering	Iron overload → Neurovascular dysfunction, neuroelectrophysiological tangles; Iron deficiency → Impaired myelin repair
Microglia	TfR1/DMT1 uptake	FPN1-mediated	Immune defense, phagocytosis, synaptic remodeling, injury repair	Overexpression of HO-1 in aging → Iron overload → Neurotoxicity, chronic inflammation

Abbreviations. TfR: ferric iron-bound transferrin. holo-Tf: ferric iron-bound transferrin. DMT: divalent metal transporter. NTBI: non-transferrin-bound iron. H-ferritin: ferritin heavy chain. Tim-1: T-cell immunoglobulin mucin domain 1 protein. FPN: ferroportin. MS: multiple sclerosis. HO: heme oxygenase. BBB: blood–brain barrier.

**Table 2:** Summary of QSM and mapping concordance across normal aging and neurological diseases.

Condition	Key QSM Findings	Progressive Mapping	[Multimodal] Biomarker Concordance	Key Uncertainties
Normal Aging	Iron accumulation in deep gray matter (e.g., SN, RN, DN, striatum) and cortical regions (motor, prefrontal, insula, visual cortices)	↑Iron in men > women after sex hormone reduction	[QSM + gene expression mapping] Concordance: QSM correlates with ferritin, transferrin, FPN1, DMT1 expression.	Whether iron shifts reflect normal aging or early neurodegeneration remains uncertain
Parkinson’s Disease (PD)	Iron accumulation in SN, N1; extended to RN, DN, other basal ganglia structures, hippocampus, insula, orbitofrontal cortex	↑Iron in SN: PD > iRBD > healthy	[QSM + DA transporter imaging] Concordance: Elevated SN iron parallels nigrostriatal dopaminergic dysfunction.	Unsolved directionality of iron and α-synuclein aggregation
Alzheimer’s Disease (AD)	Iron accumulation in pallidum, caudate, putamen, hippocampus (fimbria)	↑Iron: AD > MCI > healthy	[QSM + Aβ PET imaging] Partial concordance: Elevated iron in basal ganglia and hippocampus matches PET Aβ. Hippocampal iron predicts cognition independent of PET Aβ.	Unsolved directionality of iron and Aβ deposition
Multiple Sclerosis (MS)	Confounded susceptibility in deep gray matter	Hyperintensity in chronic inactive plaques; Rim of chronic active plaques; Hypointensity in remyelination	Uncertain concordance: Iron likely concordant with activated microglia in the cores of lesion.	Uncertain relative contribution of iron vs. demyelination
Cerebral Small Vessel Disease (CSVD)	Higher susceptibility in putamen, thalamus, hippocampus; CADASIL shows iron in putamen, caudate, and temporal pole	↑Iron as CSVD progresses	*[QSM + monogenetic mapping]* Concordance: Elevated iron in symptomatic *NOTCH*3 carrier, correlates with greater BBB disruption and cognitive decline.	Unsolved directionality of iron and BBB damage

Abbreviations. SN: substantia nigra. RN: red nucleus. DN: dentate nucleus. QSM: quantitative susceptibility mapping. DMT: divalent metal transporter. FPN: ferroportin. PD: Parkinson’s disease. iRBD: idiopathic rapid eye movement sleep behaviour disorder. DA: dopamine. AD: Alzheimer’s disease. Aβ: beta amyloid. PET: positron emitting tomography. MS: multiple sclerosis. CSVD: cerebral small vessel disease. CADASIL: cerebral autosomal dominant arteriopathy with subcortical infarcts and leukoencephalopathy. BBB: blood–brain barrier.

**Table 3:** Summary of regional QSM iron patterns across normal aging and neurological diseases.

Brain Region	Normal Aging	PD	AD	MS	CSVD
**Red nucleus (RN)**	Increase	Increase	Increase		Increase
**Dentate nucleus (DN)**	Increase	Increase	Increase		Increase
**Basal ganglia**	Robust general accumulation as ages	Robust accumulation in SN	Robust accumulation in caudate, putamen, pallidum	Increase	Increase
**Centrum semiovale**					*Increase*
**Hippocampus**		Increase in advanced PD	Increase		Increase
**Thalamus**				Inconsistent findings	Increase
**Cortex**	Increase in prefrontal, motor, insula, visual cortices	Increase in orbitofrontal cortex and insula	Increase in frontal cortex		Increase in temporal pole

Abbreviations. RN: red nucleus. DN: dentate nucleus. SN: substantia nigra. QSM: quantitative susceptibility mapping. PD: Parkinson’s disease. AD: Alzheimer’s disease. MS: multiple sclerosis. CSVD: cerebral small vessel disease.

## Data Availability

No new data were created or analyzed in this study.

## Abbreviations

The following abbreviations are used in this manuscript:

QSM	Quantitative susceptibility mapping
MRI	Magnetic resonance imaging
fMRI	Functional magnetic resonance imaging
GRE	Gradient Echo
AD	Alzheimer’s disease
PD	Parkinson’s disease
MS	Multiple sclerosis
CSVD	Cerebral small vessel disease
SWI	Susceptibility-weighted imaging
CNS	Central nervous system
holo-Tf	Ferric iron-bound transferrin
TfR	Transferrin receptor
BBB	Blood–brain barrier
STEAP	Six-transmembrane epithelial antigen of prostate
DMT	Divalent metal transporter
FPN	Ferroportin
NTBI	Non-transferrin-bound iron
Tim	T-cell immunoglobulin mucin
H-ferritin	Ferritin heavy chain
HO	Heme oxygenase
SN	Substantia nigra
RN	Red nucleus
DN	Dentate nucleus
DA	Dopamine
iRBD	Idiopathic rapid eye movement sleep behaviour disorder
SNc	Substantia nigra compacta
N1	Nigrosome 1
MCI	Mild cognitive impairment
Aβ	Beta amyloid
PET	Positron emitting tomography
BIOCARD	Biomarkers for Older Controls at Risk for Dementia
AQP-4	Aquaporin-4
WMH	White matter hyperintensity
CMB	Cerebral microbleeds
EPVS	Enlarged perivascular space
CADASIL	Cerebral autosomal dominant arteriopathy with subcortical infarcts and leukoencephalopathy
ICC	Intra-class correlation coefficient
TE	Time of echo

## References

[1] Bian C., Wan Y., Koduri S., Hua Y., Keep R.F., Xi G. (2023). Iron-Induced Hydrocephalus: The Role of Choroid Plexus Stromal Macrophages. Transl. Stroke Res..

[2] Dusek P., Hofer T., Alexander J., Roos P.M., Aaseth J.O. (2022). Cerebral Iron Deposition in Neurodegeneration. Biomolecules.

[3] Drayer B., Burger P., Darwin R., Riederer S., Herfkens R., Johnson G.A. (1986). MRI of brain iron. AJR Am. J. Roentgenol..

[4] Langkammer C., Schweser F., Krebs N., Deistung A., Goessler W., Scheurer E., Sommer K., Reishofer G., Yen K., Fazekas F. (2012). Quantitative susceptibility mapping (QSM) as a means to measure brain iron? A post mortem validation study. Neuroimage.

[5] Ramagiri S., Pan S., DeFreitas D., Yang P.H., Raval D.K., Wozniak D.F., Esakky P., Strahle J.M. (2023). Deferoxamine Prevents Neonatal Posthemorrhagic Hydrocephalus Through Choroid Plexus-Mediated Iron Clearance. Transl. Stroke Res..

[6] Qian Z.-M., Ke Y. (2019). Brain iron transport. Biol. Rev..

[7] Rouault T.A. (2013). Iron metabolism in the CNS: Implications for neurodegenerative diseases. Nat. Rev. Neurosci..

[8] Skjørringe T., Burkhart A., Johnsen K.B., Moos T. (2015). Divalent metal transporter 1 (DMT1) in the brain: Implications for a role in iron transport at the blood-brain barrier, and neuronal and glial pathology. Front. Mol. Neurosci..

[9] Vela D. (2018). Hepcidin, an emerging and important player in brain iron homeostasis. J. Transl. Med..

[10] Duck K.A., Connor J.R. (2016). Iron uptake and transport across physiological barriers. BioMetals.

[11] Vymazal J., Urgosík D., Bulte J.W. (2000). Differentiation between hemosiderin- and ferritin-bound brain iron using nuclear magnetic resonance and magnetic resonance imaging. Cell. Mol. Biol..

[12] Haque T.L., Miki Y., Kanagaki M., Takahashi T., Yamamoto A., Konishi J., Nozaki K., Hashimoto N., Konishi J. (2003). MR contrast of ferritin and hemosiderin in the brain: Comparison among gradient-echo, conventional spin-echo and fast spin-echo sequences. Eur. J. Radiol..

[13] Qian Z.-M., Li W., Guo Q. (2023). Ferroportin1 in the brain. Ageing Res. Rev..

[14] Zheng H., Guo X., Kang S., Li Z., Tian T., Li J., Wang F., Yu P., Chang S., Chang Y.-Z. (2023). Cdh5-mediated Fpn1 deletion exerts neuroprotective effects during the acute phase and inhibitory effects during the recovery phase of ischemic stroke. Cell Death Dis..

[15] Li Y.-Q., Guo C. (2021). A Review on Lactoferrin and Central Nervous System Diseases. Cells.

[16] Jäkel S., Agirre E., Mendanha Falcão A., van Bruggen D., Lee K.W., Knuesel I., Malhotra D., ffrench-Constant C., Williams A., Castelo-Branco G. (2019). Altered human oligodendrocyte heterogeneity in multiple sclerosis. Nature.

[17] Yeung M.S.Y., Djelloul M., Steiner E., Bernard S., Salehpour M., Possnert G., Brundin L., Frisén J. (2019). Dynamics of oligodendrocyte generation in multiple sclerosis. Nature.

[18] Chiou B., Lucassen E., Sather M., Kallianpur A., Connor J. (2018). Semaphorin4A and H-ferritin utilize Tim-1 on human oligodendrocytes: A novel neuro-immune axis. Glia.

[19] Todorich B., Zhang X., Connor J.R. (2011). H-ferritin is the major source of iron for oligodendrocytes. Glia.

[20] Li Y., Guan Q., Chen Y., Han H., Liu W., Nie Z. (2013). Transferrin receptor and ferritin-H are developmentally regulated in oligodendrocyte lineage cells. Neural Regen. Res..

[21] Khattar N., Triebswetter C., Kiely M., Ferrucci L., Resnick S.M., Spencer R.G., Bouhrara M. (2021). Investigation of the association between cerebral iron content and myelin content in normative aging using quantitative magnetic resonance neuroimaging. Neuroimage.

[22] Cohen Z., Lau L., Ahmed M., Jack C.R., Liu C. (2024). Quantitative susceptibility mapping in the brain reflects spatial expression of genes involved in iron homeostasis and myelination. Hum. Brain Mapp..

[23] Price B.R., Norris C.M., Sompol P., Wilcock D.M. (2018). An emerging role of astrocytes in vascular contributions to cognitive impairment and dementia (VCID). J. Neurochem..

[24] Cheli V., Santiago González D., Wan Q., Denaroso G., Wan R., Rosenblum S., Paez P. (2021). H-ferritin expression in astrocytes is necessary for proper oligodendrocyte development and myelination. Glia.

[25] You L., Yu P.-P., Dong T., Guo W., Chang S., Zheng B., Ci Y., Wang F., Yu P., Gao G. (2022). Astrocyte-derived hepcidin controls iron traffic at the blood-brain-barrier via regulating ferroportin 1 of microvascular endothelial cells. Cell Death Dis..

[26] Zimmer T.S., David B., Broekaart D.W.M., Schidlowski M., Ruffolo G., Korotkov A., van der Wel N.N., van Rijen P.C., Mühlebner A., van Hecke W. (2021). Seizure-mediated iron accumulation and dysregulated iron metabolism after status epilepticus and in temporal lobe epilepsy. Acta Neuropathol..

[27] Fernández-Mendívil C., Luengo E., Trigo-Alonso P., García-Magro N., Negredo P., López M.G. (2021). Protective role of microglial HO-1 blockade in aging: Implication of iron metabolism. Redox Biol..

[28] Song W., Cressatti M., Zukor H., Liberman A., Galindez C., Schipper H.M. (2017). Parkinsonian features in aging GFAP.HMOX1 transgenic mice overexpressing human HO-1 in the astroglial compartment. Neurobiol. Aging.

[29] Langkammer C., Krebs N., Goessler W., Scheurer E., Ebner F., Yen K., Fazekas F., Ropele S. (2010). Quantitative MR Imaging of Brain Iron: A Postmortem Validation Study. Radiology.

[30] Acosta-Cabronero J., Betts M.J., Cardenas-Blanco A., Yang S., Nestor P.J. (2016). In Vivo MRI Mapping of Brain Iron Deposition across the Adult Lifespan. J. Neurosci..

[31] Haller S., Haacke E.M., Thurnher M.M., Barkhof F. (2021). Susceptibility-weighted Imaging: Technical Essentials and Clinical Neurologic Applications. Radiology.

[32] Mitchell T., Lehéricy S., Chiu S.Y., Strafella A.P., Stoessl A.J., Vaillancourt D.E. (2021). Emerging Neuroimaging Biomarkers Across Disease Stage in Parkinson Disease: A Review. JAMA Neurol..

[33] Ciraci S., Gumus K., Doganay S., Dundar M.S., Kaya Ozcora G.D., Gorkem S.B., Per H., Coskun A. (2017). Diagnosis of intracranial calcification and hemorrhage in pediatric patients: Comparison of quantitative susceptibility mapping and phase images of susceptibility-weighted imaging. Diagn. Interv. Imaging.

[34] Kang C., Mehta P., Chang Y.S., Bhadelia R.A., Rojas R., Wintermark M., Andre J.B., Yang E., Selim M., Thomas A.J. (2025). Enhanced reader confidence and differentiation of calcification from cerebral microbleed diagnosis using QSM relative to SWI. Clin. Neuroradiol..

[35] Wang Y., Spincemaille P., Liu Z., Dimov A., Deh K., Li J., Zhang Y., Yao Y., Gillen K.M., Wilman A.H. (2017). Clinical quantitative susceptibility mapping (QSM)—Biometal imaging and its emerging roles in patient care. J. Magn. Reson. Imaging.

[36] Luo Y., Gao K., Zhou Y., Fawaz M., Mark Haacke E., Xia S., Liu S. (2025). Differentiating calcifications from cerebral microbleeds using quantitative susceptibility mapping. Eur. Radiol..

[37] Möller H.E., Bossoni L., Connor J.R., Crichton R.R., Does M.D., Ward R.J., Zecca L., Zucca F.A., Ronen I. (2019). Iron, Myelin, and the Brain: Neuroimaging Meets Neurobiology. Trends Neurosci..

[38] Madden D.J., Merenstein J.L. (2023). Quantitative susceptibility mapping of brain iron in healthy aging and cognition. NeuroImage.

[39] Filo S., Shaharabani R., Bar Hanin D., Adam M., Ben-David E., Schoffman H., Margalit N., Habib N., Shahar T., Mezer A.A. (2023). Non-invasive assessment of normal and impaired iron homeostasis in the brain. Nat. Commun..

[40] Choi J.Y., Cho H., Ahn S.J., Lee J.H., Ryu Y.H., Lee M.S., Lyoo C.H. (2018). Off-Target 18F-AV-1451 Binding in the Basal Ganglia Correlates with Age-Related Iron Accumulation. J. Nucl. Med..

[41] Ravanfar P., Loi S.M., Syeda W.T., Van Rheenen T.E., Bush A.I., Desmond P., Cropley V.L., Lane D.J.R., Opazo C.M., Moffat B.A. (2021). Systematic Review: Quantitative Susceptibility Mapping (QSM) of Brain Iron Profile in Neurodegenerative Diseases. Front. Neurosci..

[42] Burgetova R., Dusek P., Burgetova A., Pudlac A., Vaneckova M., Horakova D., Krasensky J., Varga Z., Lambert L. (2021). Age-related magnetic susceptibility changes in deep grey matter and cerebral cortex of normal young and middle-aged adults depicted by whole brain analysis. Quant. Imaging Med. Surg..

[43] Howard C.M., Jain S., Cook A.D., Packard L.E., Mullin H.A., Chen N., Liu C., Song A.W., Madden D.J. (2022). Cortical iron mediates age-related decline in fluid cognition. Hum. Brain Mapp..

[44] Stawski R.S., Almeida D.M., Lachman M.E., Tun P.A., Rosnick C.B. (2010). Fluid Cognitive Ability is associated with Greater Exposure and Smaller Emotional Reactions to Daily Stressors. Psychol. Aging.

[45] Kim M., Chang Y., Cho Y., Kwon M.-J., Joh H.-K., Lim G.-Y., Kwon R., Ahn J., Park J., Kim K.-H. (2025). Accelerated increase in ferritin levels during menopausal transition as a marker of metabolic health. Sci. Rep..

[46] Qamar K., Saboor M., Qudsia F., Khosa S.M., Moinuddin, Usman M. (2015). Malabsorption of iron as a cause of iron deficiency anemia in postmenopausal women. Pak. J. Med. Sci..

[47] Persson N., Wu J., Zhang Q., Liu T., Shen J., Bao R., Ni M., Liu T., Wang Y., Spincemaille P. (2015). Age and sex related differences in subcortical brain iron concentrations among healthy adults. Neuroimage.

[48] Grubić Kezele T., Ćurko-Cofek B. (2020). Age-related changes and sex-related differences in brain iron metabolism. Nutrients.

[49] Tolosa E., Garrido A., Scholz S.W., Poewe W. (2021). Challenges in the diagnosis of Parkinson’s disease. Lancet Neurol..

[50] Du G., Wang E., Sica C., Chen H., De Jesus S., Lewis M.M., Kong L., Connor J., Mailman R.B., Huang X. (2022). Dynamics of Nigral Iron Accumulation in Parkinson’s Disease: From Diagnosis to Late Stage. Mov. Disord..

[51] Guan X., Guo T., Zhou C., Wu J., Zeng Q., Li K., Luo X., Bai X., Wu H., Gao T. (2022). Altered brain iron depositions from aging to Parkinson’s disease and Alzheimer’s disease: A quantitative susceptibility mapping study. NeuroImage.

[52] Ward R.J., Zucca F.A., Duyn J.H., Crichton R.R., Zecca L. (2014). The role of iron in brain ageing and neurodegenerative disorders. Lancet Neurol..

[53] Dichtl S., Haschka D., Nairz M., Seifert M., Volani C., Lutz O., Weiss G. (2018). Dopamine promotes cellular iron accumulation and oxidative stress responses in macrophages. Biochem. Pharmacol..

[54] Zecca L., Bellei C., Costi P., Albertini A., Monzani E., Casella L., Gallorini M., Bergamaschi L., Moscatelli A., Turro N.J. (2008). New melanic pigments in the human brain that accumulate in aging and block environmental toxic metals. Proc. Natl. Acad. Sci. USA.

[55] Iranzo A., Santamaria J., Tolosa E. (2016). Idiopathic rapid eye movement sleep behaviour disorder: Diagnosis, management, and the need for neuroprotective interventions. Lancet Neurol..

[56] Sun J., Lai Z., Ma J., Gao L., Chen M., Chen J., Fang J., Fan Y., Bao Y., Zhang D. (2020). Quantitative Evaluation of Iron Content in Idiopathic Rapid Eye Movement Sleep Behavior Disorder. Mov. Disord..

[57] Friedrich I., Reimann K., Jankuhn S., Kirilina E., Stieler J., Sonntag M., Meijer J., Weiskopf N., Reinert T., Arendt T. (2021). Cell specific quantitative iron mapping on brain slices by immuno-µPIXE in healthy elderly and Parkinson’s disease. Acta Neuropathol. Commun..

[58] Thomas G.E.C., Hannaway N., Zarkali A., Shmueli K., Weil R.S. (2024). Longitudinal associations of magnetic susceptibility with clinical severity in parkinson’s disease. Mov. Disord..

[59] Thomas G.E.C., Leyland L.A., Schrag A.-E., Lees A.J., Acosta-Cabronero J., Weil R.S. (2020). Brain iron deposition is linked with cognitive severity in Parkinson’s disease. J. Neurol. Neurosurg. Psychiatry.

[60] Billings J.L., Gordon S.L., Rawling T., Doble P.A., Bush A.I., Adlard P.A., Finkelstein D.I., Hare D.J. (2019). l-3,4-dihydroxyphenylalanine (l-DOPA) modulates brain iron, dopaminergic neurodegeneration and motor dysfunction in iron overload and mutant alpha-synuclein mouse models of parkinson’s disease. J. Neurochem..

[61] Scheltens P., Strooper B.D., Kivipelto M., Holstege H., Chételat G., Teunissen C.E., Cummings J., van der Flier W.M. (2021). Alzheimer’s disease. Lancet.

[62] Cogswell P.M., Wiste H.J., Senjem M.L., Gunter J.L., Weigand S.D., Schwarz C.G., Arani A., Therneau T.M., Lowe V.J., Knopman D.S. (2021). Associations of quantitative susceptibility mapping with Alzheimer’s disease clinical and imaging markers. Neuroimage.

[63] Kenkhuis B., Somarakis A., de Haan L., Dzyubachyk O., IJsselsteijn M.E., de Miranda N.F.C.C., Lelieveldt B.P.F., Dijkstra J., van Roon-Mom W.M.C., Höllt T. (2021). Iron loading is a prominent feature of activated microglia in Alzheimer’s disease patients. Acta Neuropathol. Commun..

[64] Wang F., Wang J., Shen Y., Li H., Rausch W.-D., Huang X. (2022). Iron Dyshomeostasis and Ferroptosis: A New Alzheimer’s Disease Hypothesis?. Front. Aging Neurosci..

[65] Meadowcroft M.D., Connor J.R., Smith M.B., Yang Q.X. (2009). Magnetic Resonance Imaging and Histological Analysis of Beta-Amyloid Plaques in Both Human Alzheimer’s Disease and APP/PS1 Transgenic Mice. J. Magn. Reson. Imaging.

[66] Chen L., Soldan A., Oishi K., Faria A., Zhu Y., Albert M., van Zijl P.C.M., Li X. (2021). Quantitative Susceptibility Mapping of Brain Iron and β-Amyloid in MRI and PET Relating to Cognitive Performance in Cognitively Normal Older Adults. Radiology.

[67] Au C.K.F., Abrigo J., Liu C., Liu W., Lee J., Au L.W.C., Chan Q., Chen S., Leung E.Y.L., Ho C.L. (2021). Quantitative Susceptibility Mapping of the Hippocampal Fimbria in Alzheimer’s Disease. J. Magn. Reson. Imaging.

[68] Bittner S., Oh J., Havrdová E.K., Tintoré M., Zipp F. (2021). The potential of serum neurofilament as biomarker for multiple sclerosis. Brain J. Neurol..

[69] Marcus R. (2022). What Is Multiple Sclerosis?. JAMA.

[70] Ziemssen T., Akgün K., Brück W. (2019). Molecular biomarkers in multiple sclerosis. J. Neuroinflamm..

[71] Langkammer C., Liu T., Khalil M., Enzinger C., Jehna M., Fuchs S., Fazekas F., Wang Y., Ropele S. (2013). Quantitative Susceptibility Mapping in Multiple Sclerosis. Radiology.

[72] Stankiewicz J.M., Neema M., Ceccarelli A. (2014). Iron and multiple sclerosis. Neurobiol. Aging.

[73] Gillen K.M., Mubarak M., Park C., Ponath G., Zhang S., Dimov A., Levine-Ritterman M., Toro S., Huang W., Amici S. (2021). QSM is an imaging biomarker for chronic glial activation in multiple sclerosis lesions. Ann. Clin. Transl. Neurol..

[74] Rahmanzadeh R., Galbusera R., Lu P.-J., Bahn E., Weigel M., Barakovic M., Franz J., Nguyen T.D., Spincemaille P., Schiavi S. (2022). A New Advanced MRI Biomarker for Remyelinated Lesions in Multiple Sclerosis. Ann. Neurol..

[75] Stüber C., Pitt D., Wang Y. (2016). Iron in Multiple Sclerosis and Its Noninvasive Imaging with Quantitative Susceptibility Mapping. Int. J. Mol. Sci..

[76] Schoonheim M.M., Pinter D., Prouskas S.E., Broeders T.A., Pirpamer L., Khalil M., Ropele S., Uitdehaag B.M., Barkhof F., Enzinger C. (2022). Disability in multiple sclerosis is related to thalamic connectivity and cortical network atrophy. Mult. Scler..

[77] Burgetova A., Dusek P., Vaneckova M., Horakova D., Langkammer C., Krasensky J., Sobisek L., Matras P., Masek M., Seidl Z. (2017). Thalamic Iron Differentiates Primary-Progressive and Relapsing-Remitting Multiple Sclerosis. Am. J. Neuroradiol..

[78] Louapre C., Govindarajan S.T., Giannì C., Madigan N., Sloane J.A., Treaba C.A., Herranz E., Kinkel R.P., Mainero C. (2018). Heterogeneous pathological processes account for thalamic degeneration in multiple sclerosis: Insights from 7 T imaging. Mult. Scler..

[79] Zivadinov R., Tavazzi E., Bergsland N., Hagemeier J., Lin F., Dwyer M.G., Carl E., Kolb C., Hojnacki D., Ramasamy D. (2018). Brain Iron at Quantitative MRI Is Associated with Disability in Multiple Sclerosis. Radiology.

[80] Filippi M., Preziosa P., Barkhof F., Ciccarelli O., Cossarizza A., De Stefano N., Gasperini C., Geraldes R., Granziera C., Haider L. (2024). The ageing central nervous system in multiple sclerosis: The imaging perspective. Brain.

[81] Magyari M., Sorensen P.S. (2020). Comorbidity in multiple sclerosis. Front. Neurol..

[82] Inoue Y., Shue F., Bu G., Kanekiyo T. (2023). Pathophysiology and probable etiology of cerebral small vessel disease in vascular dementia and Alzheimer’s disease. Mol. Neurodegener..

[83] Uchida Y., Kan H., Sakurai K., Arai N., Inui S., Kobayashi S., Kato D., Ueki Y., Matsukawa N. (2020). Iron leakage owing to blood–brain barrier disruption in small vessel disease CADASIL. Neurology.

[84] Li J., Nguyen T.D., Zhang Q., Guo L., Wang Y. (2022). Cerebral Microbleeds Are Associated With Increased Brain Iron and Cognitive Impairment in Patients With Cerebral Small Vessel Disease: A Quantitative Susceptibility Mapping Study. J. Magn. Reson. Imaging.

[85] Perosa V., Rotta J., Yakupov R., Kuijf H.J., Schreiber F., Oltmer J.T., Mattern H., Heinze H.-J., Düzel E., Schreiber S. (2023). Implications of quantitative susceptibility mapping at 7 tesla MRI for microbleeds detection in cerebral small vessel disease. Front. Neurol..

[86] Gao Y., Liang C., Zhang Q., Zhuang H., Sui C., Zhang N., Feng M., Xin H., Guo L., Wang Y. (2025). Brain iron deposition and cognitive decline in patients with cerebral small vessel disease: A quantitative susceptibility mapping study. Alzheimer’s Res. Ther..

[87] Farr A.C., Xiong M.P. (2021). Challenges and opportunities of deferoxamine delivery for treatment of alzheimer’s disease, parkinson’s disease, and intracerebral hemorrhage. Mol. Pharm..

[88] Negida A., Hassan N.M., Aboeldahab H., Zain Y.E., Negida Y., Cadri S., Cadri N., Cloud L.J., Barrett M.J., Berman B. (2024). Efficacy of the iron-chelating agent, deferiprone, in patients with parkinson’s disease: A systematic review and meta-analysis. CNS Neurosci. Ther..

[89] Devos D., Labreuche J., Rascol O., Corvol J.-C., Duhamel A., Guyon Delannoy P., Poewe W., Compta Y., Pavese N., Růžička E. (2022). Trial of deferiprone in parkinson’s disease. N. Engl. J. Med..

[90] Ayton S., Barton D., Brew B., Brodtmann A., Clarnette R., Desmond P., Devos D., Ellis K.A., Fazlollahi A., Fradette C. (2025). Deferiprone in alzheimer disease: A randomized clinical trial. JAMA Neurol..

[91] Wang Y., Wu S., Li Q., Sun H., Wang H. (2023). Pharmacological inhibition of ferroptosis as a therapeutic target for neurodegenerative diseases and strokes. Adv. Sci..

[92] Langkammer C., Schweser F., Shmueli K., Kames C., Li X., Guo L., Milovic C., Kim J., Wei H., Bredies K. (2018). Quantitative susceptibility mapping: Report from the 2016 reconstruction challenge. Magn. Reson. Med..

[93] Bilgic B., Langkammer C., Marques J.P., Meineke J., Milovic C., Schweser F., QSM Challenge 2.0 Organization Committee (2021). QSM reconstruction challenge 2.0: Design and report of results. Magn. Reson. Med..

[94] Rua C., Clarke W.T., Driver I.D., Mougin O., Morgan A.T., Clare S., Francis S., Muir K.W., Wise R.G., Carpenter T.A. (2020). Multi-centre, multi-vendor reproducibility of 7T QSM and R2* in the human brain: Results from the UK7T study. NeuroImage.

[95] Lancione M., Bosco P., Costagli M., Nigri A., Aquino D., Carne I., Ferraro S., Giulietti G., Napolitano A., Palesi F. (2022). Multi-centre and multi-vendor reproducibility of a standardized protocol for quantitative susceptibility mapping of the human brain at 3T. Phys. Medica.

[96] Nigri A., Ferraro S., Gandini Wheeler-Kingshott C.A.M., Tosetti M., Redolfi A., Forloni G., D’Angelo E., Aquino D., Biagi L., Bosco P. (2022). Quantitative MRI harmonization to maximize clinical impact: The RIN–neuroimaging network. Front. Neurol..

[97] Bilgic B., Costagli M., Chan K.-S., Duyn J., Langkammer C., Lee J., Li X., Liu C., Marques J.P., QSM Consensus Organization Committee (2024). Recommended implementation of quantitative susceptibility mapping for clinical research in the brain: A consensus of the ISMRM electro-magnetic tissue properties study group. Magn. Reson. Med..

[98] Lancione M., Donatelli G., Cecchi P., Cosottini M., Tosetti M., Costagli M. (2019). Echo-time dependency of quantitative susceptibility mapping reproducibility at different magnetic field strengths. NeuroImage.

[99] Bordin V., Pirastru A., Bergsland N., Cazzoli M., Baselli G., Baglio F. (2023). Optimal echo times for quantitative susceptibility mapping: A test-retest study on basal ganglia and subcortical brain nuclei. NeuroImage.

[100] Liu T., Spincemaille P., de Rochefort L., Kressler B., Wang Y. (2009). Calculation of susceptibility through multiple orientation sampling (COSMOS): A method for conditioning the inverse problem from measured magnetic field map to susceptibility source image in MRI. Magn. Reson. Med..

[101] Soret M., Bacharach S.L., Buvat I. (2007). Partial-volume effect in PET tumor imaging. J. Nucl. Med..

[102] Berg R.C., Preibisch C., Thomas D.L., Shmueli K., Biondetti E. (2021). Investigating the effect of flow compensation and quantitative susceptibility mapping method on the accuracy of venous susceptibility measurement. NeuroImage.

